# Epidemiology of nursing home dialysis patients—A hidden population

**DOI:** 10.1111/hdi.12943

**Published:** 2021-06-16

**Authors:** Eran Y. Bellin, Alice M. Hellebrand, Steven M. Kaplan, Jordan G. Ledvina, William T. Markis, Nathan W. Levin, Allen M. Kaufman

**Affiliations:** ^1^ Departments of Epidemiology & Population Health and Medicine Albert Einstein College of Medicine Bronx New York USA; ^2^ Dialyze Direct Neptune City New Jersey USA; ^3^ WTM Consulting Passaic New Jersey USA; ^4^ Internal Medicine Mount Sinai Icahn School of Medicine New York New York USA

**Keywords:** epidemiology, ESRD, hemodialysis, intradialytic hypotension, mortality risk factors, nursing home

## Abstract

**Introduction:**

Dialysis patients are often discharged from hospitals to skilled nursing facilities (SNFs), but little has been published about their natural history.

**Methods:**

Using electronic medical record data, we conducted a retrospective cohort study of nursing home patients treated with in‐SNF hemodialysis from January 1, 2018 through June 20, 2020 within a dialysis organization across eight states. A dialytic episode began with the first in‐SNF dialysis and was ended by hospitalization, death, transfer, or cessation of treatment. The clinical characteristics and natural history of these patients and their dialytic episodes are described.

**Findings:**

Four thousand five hundred and ten patients experienced 9274 dialytic episodes. Dialytic episodes had a median duration of 18 days (IQR: 8–38) and were terminated by a hospitalization *n* = 5747 (62%), transfer *n* = 2638 (28%), death *n* = 568 (6%), dialysis withdrawal *n* = 129 (1.4%), recovered function *n* = 2 (0.02%), or other cause *n* = 6 (0.06%). Increased patient mortality was associated with advancing age, low serum creatinine, albumin, or sodium, and low pre‐dialytic systolic blood pressure (sBP). U‐shaped relationships to mortality were observed for intradialytic hypotension frequency and for post‐ > pre‐hemodialysis sBP frequency. Prescription of dialysis five times weekly in the first 2 weeks was associated with better survival in the first 90 days (HR 0.77, CI 0.62–0.96; *p* < 0.02).

**Discussion:**

Provision of in‐SNF dialysis by an external dialysis organization enables discharge from the acute care setting for appropriate treatment with increased nursing contact time in an otherwise under‐resourced environment. SNF ESRD patient clinical characteristics and outcomes are extensively characterized for the first time.

## INTRODUCTION

Patients over 65 comprise 52% of annual US incident dialysis patients.^1^ In 2015–2017, reportedly 6.9% of incident ESRD patients resided in a skilled nursing facility (SNF)^2^—a likely underestimate.^3^ In the year preceding death, over one‐third of medicare dialysis patients resided in a SNF.^4^ However, the medical literature is virtually blind to SNF dialysis residents.^3^, ^5^, ^6^, ^7^ The latest comprehensive review summarizes reports in the decade preceding 2010.^8^ We report on our experience with this significant and growing population of hidden patients.

## MATERIALS AND METHODS

### Setting

Dialyze Direct provides onsite dialysis within SNFs, implementing a shorter and more frequent hemodialysis (MFD) treatment paradigm pursuant to a physician's order, using NxStage System One technology and modeled to deliver 14 total hours of therapy per week. With a centralized administrative structure and distributed model of care (Figures S1–S3), senior clinical leadership develops protocols, procedures, and layered supervision across 134 nursing homes in eight states (Illinois, Ohio, Florida, Texas, New York, Pennsylvania, Indiana, Maryland). We report a retrospective study of all dialysis care data recorded by Dialyze Direct from January 1, 2018 through June 20, 2020 in either of two electronic medical records (EMR), GAIA (Gaia, Littleton, CO) and Clarity (Visonex, Green Bay, WI). This study adhered to the principles outlined in the Declaration of Helsinki. The Institutional Review Board (Western IRB, Olympia, WA) ruled the protocol exempt with full waiver of informed consent under 45 CFR § 46.104(d)^2^ of the Common Rule.

### Analytical construct: Dialytic episode

The dialytic episode was defined as the interval starting from the first in‐nursing home dialysis session, inclusive of all subsequent dialysis sessions, until terminated by hospitalization, transfer to another facility, death, withdrawal from therapy, or transfer to home. In readmission to the SNF, the first dialysis session upon readmission initiates a new dialytic episode. We report only completed dialytic episodes, excluding those beginning but not terminated within the timeframe of our study. For a given patient, an event life‐history is created by combining these dialytic episodes, allowing for multiple hospitalizations in follow‐up. Censorship is established by the last nondeath dialytic episode terminating event. In this framework, each dialytic episode can encompass many dialysis sessions and provide detailed insights into patient outcomes. Tabular data are provided by dialytic episode, representing every patient‐admission to the SNF, or by patient, using only the first SNF admission.

Systolic blood pressures (sBP) pre‐ and post‐hemodialysis (HD) recorded during standard care delivery were separately averaged across all dialyses within a dialytic episode to provide average values for each episode. Intradialytic hypotension (IDH) is defined according to Flythe with modification^9^, ^10^, ^11^ using a threshold absolute nadir sBP during a dialysis session. IDH is recognized by an absolute nadir sBP <90 mmHg, or alternatively, <100 mmHg in the case of a pre‐HD sBP ≥150 mmHg. For each dialytic episode, we calculate the percent of dialyses in which at least one IDH event occurred and study its relationship to mortality. We also repeated this analysis for sBP <85 mmHg.

Peridialytic sBP changes were calculated as post‐HD sBP minus pre‐HD sBP and assessed for frequency per dialytic episode of positive peridialytic sBP (post‐HD sBP > pre‐HD sBP, by at least 5 mmHg).

Dialytic kinetic adequacy is defined as delivery of standard *Kt*/*V* (std*Kt*/*V*) ≥2.0. Kinetics are measured at first HD and then repeatedly on the first Monday of each month with subsequent adjustments in the dialysis prescription as needed to achieve adequacy. Kinetic calculations are made using the formula developed by Leypoldt.^12^ Kinetic data are presented as the average std*Kt*/*V* per dialytic episode.

The race/ethnicity variable was constructed sequentially by first assigning ethnicity and then delineating the non‐Hispanic population as White, Black, or other/unknown.

All‐cause mortality information is obtained from EMR and includes death in the nursing home as well as in the hospital when known.

Duration of dialysis is obtained from the vintage field in the EMR where there is considerable incompleteness and obvious errors, so we present this information as median and interquartile range. We attempt to calculate a lower limit estimate of incidence by including all unique patients in the denominator and all patients whose vintage calculates to zero at the time of first dialytic episode in the numerator.

### Home discharge

Nurses administering in‐nursing home dialysis perceived themselves as providing in‐home dialysis, and within their EMR vocabulary, even when patients were transferred back to their original domicile, they considered them conceptually “transferred to another dialysis unit.” Their entries for disposition in the EMR reflected this mindset and thus did not capture a distinction between true home discharge vs. transfer to another facility. Beginning in April 2020, we established a process of reeducation and asked the nurses to explicitly designate discharge to home domicile in the disposition table.

### Statistical method

Stata 16 (College Park, TX) was used for data management, descriptive statistics, and time to outcome analyses, including Kaplan–Meier survival statistics and graphs combining follow‐up dialytic episodes with gaps to create a continuous event history to death or censorship for each patient.^13^ Multivariable survival analysis was performed with Cox Regression and proportionality assumption tested with Schoenfeld residuals. Associations are represented by hazard ratios (HR) and 95% confidence intervals (CI). Laboratory tests used for modeling comprise the first measure obtained in a dialytic episode.

Summary descriptions are provided as a modified Tukey five‐point data summary (FPDS)^14^ with percentiles 5, 25, 50, 75, 95 provided. This exclusion of extremes prevents inferential error due to gross database outliers.^14^ In tables, missing data is implied by differing counts of relevant constituent observations. Aggregation of problem list diagnostic codes into Elixhauser and Charlson diagnostic categories^15^, ^16^, ^17^, ^18^ was performed using the R library comorbidity.^19^, ^20^

### Intent and success in achieving 5×/week dialysis

Five times per week dialysis (MFD5) benefits patients with a gentler process requiring lower‐volume ultrafiltration, which should yield shorter post‐dialysis recovery times. Because Dialyze Direct's patient population is elderly and frail, the majority of these patients suffer from certain co‐morbidities that result in the local nephrologist of record prescribing MFD5. To evaluate achievement of this clinical goal, we define intent to treat by averaging dialysis prescriptions in the first two Sunday‐through‐Saturday intervals. We compare this calculated intent with the observed dialyses averaged over complete Sunday‐through‐Saturday intervals for the first 90 days of an episode. To evaluate association of MFD5 intent with survival, we apply the intent to treat paradigm for outcome attribution, assessing survival in the time period following established intent.

## RESULTS

### Dialytic episode outcomes

From January 1, 2018 through June 20, 2020, a total of 4510 patients initiated and completed 9274 in‐nursing home dialytic episodes under Dialyze Direct's care. Demographics and primary dialysis access are provided (Table 1).

**TABLE 1:** Demographics for first nursing home admission, dialytic episodes

Characteristics	First admission *n* = 4510	Dialytic episodes *n* = 9274
Race/ethnicity		
White	1778 (39)	3652 (39)
Black	1521 (34)	3593 (39)
Hispanic	225 (5)	487 (5)
Other/unknown	986 (22)	1542 (17)
Age: mean (SD)	69 (12.4)	68 (12.4)
Age by Category, *n* (%)		
0–50	5 (7)	737 (8)
50–60	640 (14)	1488 (16)
60–70	1273 (28)	2684 (29)
70–80	1433 (32)	2842 (31)
>80	849 (19)	1523 (16)
Gender		
Male	2340 (52)	4702 (51)
Female	2170 (48)	4572 (49)
Primary vascular access		
AV fistula—upper extremity	1520 (34)	2999 (32)
Fistula—lower extremity	7 (0.2)	11 (0.1)
AV graft—upper extremity	433 (10)	964 (10)
Graft—lower extremity	15 (0.3)	26 (0.3)
Catheter tunnel neck	2454 (54)	5034 (54)
Tunnel groin	14 (0.3)	44 (0.5)
Neck (temporary)	40 (0.9)	82 (0.9)
Groin (temporary)	22 (0.5)	93 (1)
Unknown	5 (0.1)	11 (0.1)
Vintage years (*n*)	*n* = 3564	*n* = 7839
Median (IQR) years	1.9 (0.1–5.1)	2.1 (0.4–5.3)

*Note*: Data are presented as *n* (%) unless otherwise indicated. IQR, interquartile range.

Dialytic episodes were terminated by a hospitalization *n* = 5747 (62%), transfer *n* = 2638 (28%), death *n* = 568 (6%), dialysis withdrawal *n* = 129 (1.4%), recovered function *n* = 2 (0.02%), or unspecified other cause *n* = 6 (0.06%). An additional 492 patients were known to have subsequently died during their hospitalization.

Four thousand four hundred and twenty hospitalizations were followed by a readmission to the same SNF. The median intervening duration of hospitalization was 6 days (IQR: 4–10).

One thousand eight hundred and sixty‐six patients were readmitted to the SNF following a hospitalization. These readmitted patients had a median of three hospitalizations with SNF readmission (FPDS: 2, 2, 3, 4, 8).

The median duration of dialytic episode was 18 days (IQR: 8–38). Forty‐three percent of episodes consisted of a length of stay ≤2 weeks, with 10% staying 81 days or more.

Of the 4021 dialytic episodes discharged within 2 weeks, 2737 (68%) were hospitalized, 275 (7%) died, 945 (23.5%) were transferred out, 62 (2%) had dialysis withdrawn, and 2 (0.05%) were classified as “other.” An additional 236 of those hospitalized subsequently died.

Home discharge was evaluated following improved EMR protocol (see Methods) for the period May 1, 2020 until June 20, 2020. Of 546 qualifying dialytic episodes, 98 (18%) were discharged to home, 345 (63%) hospitalized, 74 (14%) died, 14 (2.6%) transferred to SNF, 9 (1.7%) had dialysis withdrawn, 1 (0.2%) recovered function, and 5 (0.9%) designated as “other.” Of 216 discharged patients with length of stay ≤2 weeks, 149 (69%) were hospitalized, 27 (13%) died, 25 (12%) returned home, 7 (3%) had dialysis withdrawn, 6 (3%) transferred to SNF, and 2 (0.9%) were “other.”

### Clinical observations

#### Prevalence of documented clinical disease

Nursing documentation of clinical disease in the problem list section of the EMR suffers from incompleteness generally seen in most medical centers^21^ with 2315/9274 (25%) dialytic episodes failing to record even one clinical problem. Notably, even when the problem list contained at least one entry, 1246/6959 (18%) did not designate a recognizable code for renal disease. With this proviso, we provide prevalent diseases documented in problem lists (Table 2). Weighted Charlson score (without age) for dialytic episodes was median 3 (IQR: 2–5) with 10% ≥6.

**TABLE 2:** Problem list documented disease prevalence by first admission/dialytic episode (Elixhauser)

	First admission *n* = 3181	Dialytic episode *n* = 6959	ICD 10
Congestive heart failure	940 (30)	2184 (31)	I09.9, I11.0, I13.0, I13.2, I25.5, I42.0, I42.5–I42.9, I43.x, I50.x, P29.0
Cardiac arrhythmias	533 (17)	1179 (17)	I44.1 ‐ I44.3, I45.6, I45.9, I47.x ‐ I49.x, R00.0, R00.1, R00.8, T82.1, Z45.0, Z95.0
Valvular disease	87 (3)	188 (2.7)	A52.0, I05.x–I08.x, I09.1, I09.8, I34.x–I39.x, Q23.0–Q23.3, Z95.2–Z95.4
Pulmonary circulation disorder	73 (2)	176 (2.5)	I26.x, I27.x, I28.0, I28.8, I28.9
Peripheral vascular disorder	290 (9)	706 (10)	I70.x, I71.x, I73.1, I73.8, I73.9, I77.1, I79.0, I79.2, K55.1, K55.8, K55.9, Z95.8, Z95.9
Hypertension uncomplicated	1111 (35)	2572 (37)	I10.x
Hypertension complicated	1174 (37)	2931 (42)	I11.x–I13.x, I15.x
Paralysis	20 (0.6)	74 (1.1)	G04.1, G11.4, G80.1, G80.2, G81.x, G82.x, G83.0–G83.4, G83.9
Other neurologic disorders	181 (5.7)	574 (8.2)	G10.x–G13.x, G20.x–G22.x, G25.4, G25.5, G31.2, G31.8, G31.9, G32.x, G35.x–G37.x, G40.x, G41.x, G93.1, G93.4, R47.0, R56.x
Chronic pulmonary disease	427 (13.4)	1142 (16.4)	I27.8, I27.9, J40.x–J47.x, J60.x–J67.x, J68.4, J70.1, J70.3
Diabetes uncomplicated	512 (16)	1351 (19.4)	E10.0, E10.1, E10.9, E11.0, E11.1, E11.9, E12.0, E12.1, E12.9, E13.0, E13.1, E13.9, E14.0, E14.1, E14.9
Diabetes complicated	1163 (37)	2569 (37)	E10.2–E10.8, E11.2–E11.8, E12.2–E12.8, E13.2–E13.8, E14.2–E14.8
Hypothyroidism	205 (6.4)	470 (6.7)	E00.x–E03.x, E89.0
Renal failure	2687 (84)	5713 (82)	I12.0, I13.1, N18.x, N19.x, N25.0, Z49.0–Z49.2, Z94.0, Z99.2
Liver disease	116 (3.6)	364 (5.2)	B18.x, I85.x, I86.4, I98.2, K70.x, K71.1, K71.3–K71.5, K71.7, K72.x–K74.x, K76.0, K76.2–K76.9, Z94.4
Peptic ulcer disease	7 (0.2)	17 (0.2)	K25.7, K25.9, K26.7, K26.9, K27.7, K27.9, K28.7, K28.9
Aids/HIV	22 (0.7)	58 (0.8)	B20.x–B22.x, B24.x
Lymphoma	21 (0.7)	49 (0.7)	C81.x–C85.x, C88.x, C96.x, C90.0, C90.2
Metastatic cancer	11 (0.3)	20 (0.3)	C77.x–C80.x
Solid tumor without metastasis	77 (2.4)	206 (3)	C00.x–C26.x, C30.x–C34.x, C37.x–C41.x, C43.x, C45.x–C58.x, C60.x–C76.x, C97.x
Rheumatoid arthritis/collagen vasc disease	53 (1.7)	165 (2.4)	L94.0, L94.1, L94.3, M05.x, M06.x, M08.x, M12.0, M12.3, M30.x, M31.0–M31.3, M32.x–M35.x, M45.x, M46.1, M46.8, M46.9
Coagulopathy	116 (3.6)	281 (4)	D65–D68.x, D69.1, D69.3–D69.6
Obesity	176 (5.5)	472 (6.8)	E66.x
Weight loss	111 (3.5)	365 (5.2)	E40.x–E46.x, R63.4, R64
Fluid and electrolyte disorders	1854 (58)	4165 (60)	E22.2, E86.x, E87.x
Blood loss anemia	10 (0.3)	44 (0.6)	D50.0
Deficiency anemia	1046 (33)	2685 (39)	D50.8, D50.9, D51.x–D53.x
Alcohol abuse	41 (1.3)	104 (1.5)	F10, E52, G62.1, I42.6, K29.2, K70.0, K70.3, K70.9, T51.x, Z50.2, Z71.4, Z72.1
Drug abuse	34 (1.1)	110 (1.6)	F11.x–F16.x, F18.x, F19.x, Z71.5, Z72.2
Psychoses	49 (1.5)	124 (1.8)	F20.x, F22.x–F25.x, F28.x, F29.x, F30.2, F31.2, F31.5
Depression	287 (9)	803 (11.5)	F20.4, F31.3–F31.5, F32.x, F33.x, F34.1, F41.2, F43.2

*Note*: Data are presented as *n* (%).

First laboratory values for patients' first in‐SNF dialysis under our care (Table S1) and for all recorded dialytic episodes (Table S2) are summarized in percentiles.

#### Blood pressure and volume observations

Median averaged pre‐dialysis sBP was 128 mmHg (IQR: 115–144) with 10% having an averaged pre‐dialysis sBP of at least 158 mmHg. (*n* = 9139). Median averaged post‐dialysis sBP was 127 mmHg (IQR: 115–141) with 10% having an averaged post‐dialysis sBP of at least 154 mmHg. (*n* = 9129).

Median volume decrease (net of all inputs and outputs) was 0.88 L per session (IQR: 0.53–1.3). Median time on dialysis was 170 min (IQR: 150–182). Median ultrafiltration rate for patients prescribed five times weekly dialysis was 4.9 ml/h/kg (IQR: 3.1–6.9) (UFR, sp*Kt*/*V*, std*Kt*/*V* by prescribed dialysis frequency, Table 3).

**TABLE 3:** Ultrafiltration rate, sp*Kt*/*V*, and std*Kt*/*V* by dialysis prescription for *n* patient‐weeks reporting

Prescribed dialysis sessions per week	Ultrafiltration rate (ml/h/kg)	sp*Kt*/*V*	std*Kt*/*V*
3	6.8 (4.5–9.4)*n* = 8411	1.5 (1.3–1.9)*n* = 2586	
4	4.7 (2.8–6.7)*n* = 5492		2.1 (1.9–2.3)*n* = 2.051
5	4.9 (3.1–6.9)*n* = 34,323		2.2 (2.0–2.4)*n* = 12,130

*Note*: Data are presented as median (interquartile range).

We have IDH data on 9139 of 9274 dialytic episodes. Fifty percent of these dialytic episodes manifested an IDH event in at least 14% of their dialysis sessions. Ten percent of dialytic episodes manifested an IDH event in at least 75% of their dialysis sessions.

We have IDH data from 4448 of 4510 patient first SNF admissions. Fifty percent of patients experienced an IDH event in at least 14% of their dialysis sessions. Ten percent of patients experienced an IDH event in at least 75% of their dialysis sessions.

Evaluating nadir sBP <85 mmHg, 25% of patients had at least 20% of their sessions characterized as such, and 10% of patients were characterized by such a nadir in at least 50% of their dialysis sessions.

#### Intent and success in achieving 5×/week dialysis (MFD5)

Forty‐three percent of dialytic episodes lack sufficient length of stay (≥14 days) to establish intent. Of the remaining 4669 dialytic episodes, 3339 (72%) were prescribed MFD5. Of those 3339 with demonstrated MFD5 intent, 1438 (43%) achieved consistent MFD5 treatment, 1149 (34%) achieved an average of 4.5–5 days per week dialysis, and 260 (7.8%) achieved 4.1–4.5 days per week dialysis. Of those with intent <5× per week, only 66/1330 (5%) received an average dialysis frequency > 4× per week in the first 90 days.

#### Survival

Following patients from first admission until death in nursing home or hospital demonstrated a strong effect of increasing age on cumulative mortality (Logrank; *p* < 0.0001).

**FIGURE 1:**
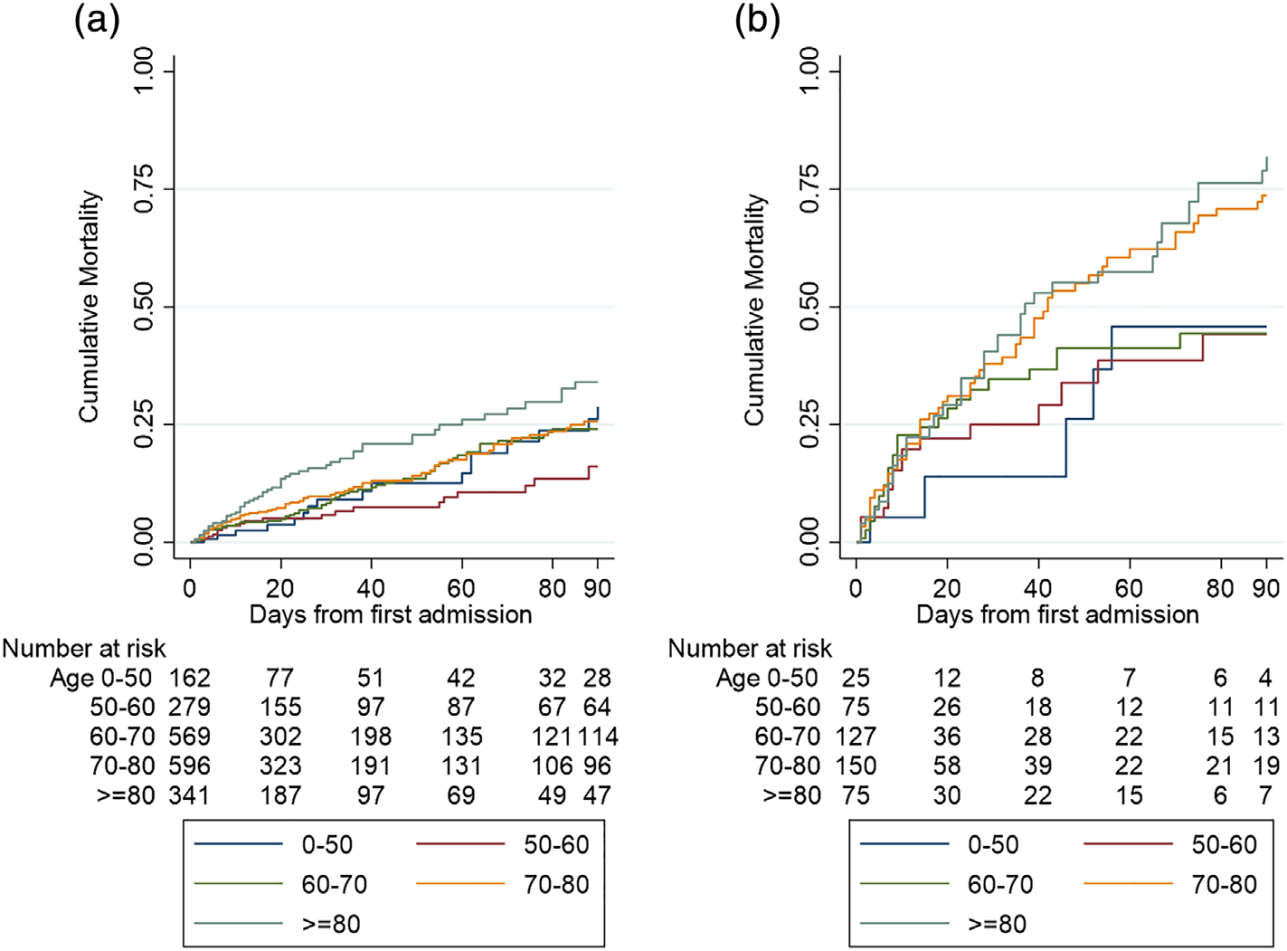
Cumulative mortality (one minus Kaplan–Meier) plots by age bracket, stratified by percent of dialyses with intradialytic hypotension within a dialytic episode. (a) 10%–25% of dialysis sessions with intradialytic hypotension and (b) 75%–100% of dialysis sessions with intradialytic hypotension. Intradialytic hypotension was defined by experiencing nadir systolic blood pressure < 90 mmHg (or <100 mmHg if pre‐hemodialysis systolic blood pressure was >150 mmHg) [Color figure can be viewed at wileyonlinelibrary.com]

Repeating the age‐mortality analysis across two strata of IDH frequency (10%–25% vs. 75%–100%) visually demonstrates the impact of the IDH metric on survival (Figure 1).

A Cox proportional hazards model (Table 4) shows increased mortality with increasing age, decreasing serum sodium, albumin, creatinine concentrations, and lower pre‐HD sBP. The lowest quintile of percentage dialyses with post‐HD sBP > pre‐HD sBP (0%–14.3%) has higher mortality compared with the highest quintile (>60%) (HR 1.5, CI [1.2–1.9]; *p* < 0.001) (different baseline than shown in Table 4).

**TABLE 4:** Cox proportional hazards model for mortality adjusting for all other variables

					Percentile values within category				
	HR	CI	*p*‐value	*N* ^b^ (dialytic episodes)	5th	25th	50th	75th	95th
Age									
0–50	Base			737	30	38	44	48	50
50–60	0.96	(0.7, 1.3)	0.8	1488	51	54	56	58	60
60–70	1.1	(0.8, 1.5)	0.5	2684	61	63	66	68	69
**70–80**	**1.5**	**(1.1, 2.0)**	**0.006**	2842	71	72	74	77	79
**≥80**	**1.9**	**(1.4, 2.6)**	**0.000**	1523	80	82	84	88	91
% IDH^a^ per dialytic Episode									
**0%–10%**	**1.6**	**(1.3, 2.0)**	**0.000**	4009	0	0	0	2%	8.3%
10%–25%	Base			1546	10%	13%	16%	20%	23%
**25%–40%**	**1.6**	**(1.3, 2.1)**	**0.000**	1126	25%	27%	31%	33%	38%
**40%–75%**	**1.9**	**(1.5, 2.3)**	**0.000**	1500	40%	47%	50%	62%	71%
**75%–100%**	**3.7**	**(2.8, 4.7)**	**0.000**	958	75%	83%	100%	100%	100%
Sodium (mg/dl)									
**0–130**	**1.5**	**(1.2, 1.8)**	**0.000**	748	122	126	128	129	129
130–135	1.1	(0.9,1.2)	0.3	2879	130	131	133	134	134
≥135	Base			5455	135	136	138	141	142
Albumin (g/dl)									
**0–2.7**	**2.9**	**(2.4, 3.6)**	**0.000**	1930	1.8	2.2	2.4	2.5	2.6
**2.7–3.1**	**1.9**	**(1.6, 2.4)**	**0.000**	2826	2.7	2.8	2.9	3	3.1
**3.1–3.5**	**1.4**	**(1.1, 1.8)**	**0.001**	2005	3.2	3.2	3.3	3.4	3.4
≥3.5	Base			2420	3.5	3.6	3.7	3.9	4.2
AvgBPsysPreHD (mmHg)									
**72–111.5**	**1.8**	**(1.4, 2.3)**	**0.000**	1827	90	99	104.5	108	111
**111.5–122.5**	**1.4**	**(1.2, 1.8)**	**0.004**	1828	112	114	117	120	122
**122.5–133.5**	**1.3**	**(1.1, 1.7)**	**0.012**	1827	123	125	128	131	133
133.5–147.8	0.86	(0.67, 1.1)	0.203	1828	134	137	140	144	147
≥147.8	Base			1829	149	152	158	167	184
BPsysPost > Pre (%)									
**0%–14.3%**	**1.96**	**(1.6, 2.4)**	**0.000**	1758	0%	0%	0%	8%	13%
14.3%–30%	Base			1882	14%	18%	22%	25%	29%
30%–44%	1	(0.83,1.2)	0.9	1809	31%	33%	36%	40%	43%
44%–60%	0.96	(0.79, 1.2)	0.8	1697	44%	50%	50%	54%	58%
**>60%**	**1.3**	**(1.1, 1.6)**	**0.01**	1979	60%	67%	73%	93%	100%
Creatinine (mg/dl)									
**0–3.7**	**1.3**	**(1.03, 1.6)**	**0.030**	1839	1.7	2.5	3	3.3	3.6
**3.7–4.9**	**1.3**	**(1.05, 1.7)**	**0.016**	1837	3.7	4	4.3	4.6	4.9
4.9–6.2	1.2	(0.93, 1.5)	0.17	1831	5	5.2	5.6	5.8	6.1
6.2–8	0.99	(0.79, 1.3)	0.99	1841	6.2	6.5	7	7.4	7.8
≥8	Base			1847	8	8.6	9.4	10.7	13.4
Gender									
Female	Base								
Male	1.01	(0.89, 1.2)	0.8						
Race/ethnicity									
White	Base								
Black	1.1	(0.96, 1.3)	0.15						
Hispanic	0.97	(0.72, 1.3)	0.88						
Other/unknown	**1.5**	**(1.3, 1.8)**	**0.000**						

*Note*: Bold denotes statistically significant, *p*‐value <0.05.

Abbreviations: BPsys, systolic blood pressure; CI, 95% confidence interval; HR, hazard ratio; IDH, intradialytic hypotension; Pre‐HD, pre‐hemodialysis.

a Percent of dialyses during a nursing home stay with at least one episode of intradialytic hypotension.

b Dialytic episodes: 9274 evaluable episodes reduced by missingness of recorded values.

Mortality results associated with percentage of dialysis sessions with IDH are counterintuitive, showing a U‐shape around the base level of the second‐lowest quintile IDH frequency. The lowest level has higher mortality than the base second‐lowest level of IDH (HR 1.6, CI [1.3–2.0]; *p* < 0.000). Higher IDH percent levels show the expected increase in mortality (HR 1.6, 1.9, 3.7; *p* = 0.000, for levels 3, 4, and 5, respectively).

Increasing IDH quintiles are inversely related to pre‐HD average sBP with a −0.47 linear regression coefficient (CI [−0.49, −0.45]; *p* = 0.000) for IDH quintiles regressed on quintiles of pre‐HD average sBP (Table 5). A sensitivity analysis using Cox proportional hazards model stratifying by quintiles of pre‐HD average sBP continues to demonstrate the U‐shaped relationship of IDH frequency to mortality for each of the lower two quintiles of pre‐HD average sBP (Tables S3 and S4).

**TABLE 5:** Percentiles of averaged pre‐hemodialysis systolic blood pressure stratified by quintiles of % intradialytic hypotension

Quintile % IDH^a^ per dialytic episode	*N*	Percentile of averaged pre‐HD systolic BP				
		5th	25th	Median	75th	95th
0%–10%	4009	110	124	137	151	173
10%–25%	1546	108	120	130	143	163
25%–40%	1126	103	114	124	139	163
40%–75%	1500	98	107	116	129	153
75%–100%	958	85	97	105	118	150
Total	9139	99	115	128	144	167

*Note*: Blood pressures expressed in mmHg.

Abbreviations: BP, blood pressure; HD, hemodialysis.

a Percent of dialyses during a nursing home stay with at least one episode of intradialytic hypotension.

Neither gender nor defined race/ethnicity had a significant impact on survival.

Proportional hazard was not violated (*p* = 0.45).

Intent to treat MFD5 was associated with significantly increased survival in the first 90 days of a dialytic episode (Logrank *p* = 0.035). A cox proportional hazards model utilizing the variables described above indicated MFD5 was protective (HR 0.83, CI [0.74–0.92]; *p* = 0.001) (proportionality not rejected; *p* = 0.83). The actual weekly treatment received by those with MFD5 intent during the first 90 days of SNF residence was a mean of 4.6 treatments compared with 3.3 for those without MFD5 intent (mean difference 1.27, CI [1.23–1.31]; *p* = 0.000).

The last tenth of the study observation period overlaps with the onset of the COVID‐19 pandemic's outbreak in the United States. COVID‐19 testing in nursing homes was limited and death certificates were unavailable to us, but we can infer a significant impact of the epidemic from mortality analyses (Figure S4).

Nursing home context and service contact time is detailed (Figure S5).

## DISCUSSION

We describe a heretofore hidden nursing home dialysis population and their natural history in terms of length of stay, hospitalization, return to home, and death.

The average ultrafiltration rate (UFR) in thrice weekly outpatient dialysis was previously reported as 10 ml/h/kg across thousands of patients within a large dialysis organization.^22^ Patients in our population with MFD5 prescription had a remarkable median UFR of 4.9 ml/h/kg (IQR: 3.1–6.9), highlighting exceptional management of volume removal with this mode of treatment. This treatment paradigm potentially averts high UFR‐associated mortality.^23^

In our patient population, reduced UFR cannot be explained simply by increased dialysis frequency. Reduced access to discretionary salt and water in the nursing home population could partially explain a lower UFR. In addition, it is possible that these vulnerable and frail patients, among the most susceptible to the punishing effects of intradialytic volume shifts, experience a reset of avidity for salt and water when released from repeated systemic trauma associated with higher UFR and more aggressive volume shifts of 3 days weekly dialysis.^24^, ^25^, ^26^

Our admission std*Kt*/*V* goal is ≥2, and we achieved std*Kt*/*V* median 2.2 (IQR: 2–2.4). At the fifth percentile of dialytic episodes, we note a low std*Kt*/*V* of 1.64. Review of the data revealed that these low values are found in patients with short length of stay, with early hospital returns precluding the protocol‐driven adjustments we utilize for optimization with longer dwelling patients.

In our population, low blood pressure is associated with increased mortality—a fact recognized in other HD populations.^27^, ^28^, ^29^, ^30^, ^31^, ^32^, ^33^, ^34^, ^35^, ^36^ A number of explanations have been proposed to explain this traditional risk factor paradox.^36^, ^37^ We believe this statistical association in our patient population is a marker of pathology, in contrast to the causal relationship in the reverse direction as traditionally understood for much of the general population. The lowest quintile pre‐HD sBP in our population is extraordinarily low (FPDS: 90, 99, 104.5, 108, 110.7). We suspect that this low sBP is indicative of a significant deterioration from a prior healthier stage in the lives of these patients when their baseline sBP was higher. Thus, their low pre‐dialysis sBP is a marker of frailty reflective of poor cardiac output and/or advanced autonomic dysfunction with impaired sympathetic response^38^ and increased risk of death.

In some studies sBP tested in outpatient dialysis centers has not shown a relationship to mortality, in contrast to ambulatory home blood pressure evaluations.^39^, ^40^ It is possible that a nursing home pre‐dialysis sBP measurement is more akin to an at‐home measurement without confounders observed in the dialysis center setting.

In the general dialysis population, a rise in peridialytic sBP is believed indicative of inadequate dialysis fluid management with residual volume overload.^9^, ^11^, ^22^ This produces an unacceptable time‐averaged blood pressure that is expected to ultimately result in poor outcomes. In our patient population, those experiencing the highest frequency (>60%) of increased post‐HD sBP have a greater hazard of death compared to second‐lowest quintile baseline (14.3%–30%) (HR 1.3, CI [1.1–1.6]; *p* = 0.012). However, paradoxically, those patients with the lowest frequency of elevated post‐HD sBP (0%–14.3%) also have a greater risk of death compared to baseline (HR 1.96, CI [1.6–2.4]; *p* = 0.000) (Table 4). A comparison using the highest quintile as baseline reveals that the lowest quintile has lower survival (HR 1.5, CI [1.2–1.9]; *p* = 0.000). The inability to occasionally manifest increased post‐HD sBP likely reflects cardiac and/or autonomic frailty.

Similarly, we observe a U‐shaped impact of intradialytic hypotension with the best survival at the second‐lowest level of percent IDH (10%–25%). Higher and lower frequencies of IDH have statistically significantly worse outcomes (Table 4). The relationship of high frequency of IDH and high mortality seems straightforward. Poor cardiac function and poor autonomic reserve is indicative of a state of frailty and carries the expectation of frequent hypotension when stressed by the volume removal of dialysis, resulting in high mortality due to this state of frailty. By this logic, we would therefore expect the lowest level of percent IDH, reflective of the least frail, to have the least mortality, yet we note that the lowest level (0%–10%) has statistically worse mortality than the second‐lowest level (10%–25%) (HR 1.6, CI [1.3–2.0]; *p* = 0.000).

A potential explanation beyond absolute IDH frequency accounts for a “dialysis practitioner chagrin response” to observed hypotension and its functional selection of the frailest for the lowest quintile. It is possible that in response to observing IDH events in a patient, a practitioner dramatically reduces their efforts at volume removal, attempting to eliminate all IDH. Successfully eliminating IDH would leave an already compromised patient with increased post‐dialysis volume, thereby unwittingly increasing mortality risk.

In this scenario, exposing the patient to some degree of IDH risk, within a certain frequency threshold, may maximize dialysis benefit. We intend to explore this hypothesis with future detailed analyses.

Low pre‐dialytic serum creatinine reflects lower muscle mass, and it as well as low serum albumin indicate malnutrition or inanition. These were as expected^41^, ^42^ associated with high mortality. Hyponatremia as reported elsewhere^43^ was also associated with mortality. The mechanism of hyponatremia in the elderly dialysis patient population is related but not limited to dilutional retention of hypotonic fluid and/or nonosmotic skin and muscle compartmentalization of sodium, the latter possibly related to potassium depletion,^44^ or other cell membrane abnormalities.^45^, ^46^, ^47^

In the 1990s in an effort to encourage the care of dialysis patients in SNFs, the National Kidney Foundation created regional workshops to foster interactions between nursing homes and dialysis staff.^48^ Rapid turnover of nursing home staff defeated the project. A sustainable program must have procedural consistency, economies of scale across many institutions, and clear benefits to all stakeholders. The Dialyze Direct approach amortizes costs and supervision across nursing homes and geographic regions, providing a stable, expert workforce to manage these complex patients with dedicated care.

It is anticipated that onsite SNF dialysis will continue to grow because of value alignment among the four stakeholders—hospital, nursing home, patient, and payor (federal government). Onsite SNF dialysis enables the hospital to discharge patients earlier, reducing their length of stay and reserving space for those requiring acute care intervention. The nursing home is enabled to accept more complex patients and benefit financially from better reimbursement without needing to invest in a complex staffing infrastructure. Onsite dialysis also frees the SNF from the cost and resource burden of transportation to local dialysis centers and the disruption of rehabilitation programs. For the patient, the SNF provides an appropriate alternative setting when acute care is no longer needed. The hospital routine of frequent vital signs monitoring and off‐hour disruptions that no longer add value can now be replaced by the more relaxed pace of the SNF. Onsite dialysis also frees the patient from disruptive transportation and interruption of physical therapy and rehabilitation. We observed a lower risk of death in the first 90 days of dialytic episode for those with MFD5 intent, which points to possible outcome benefit from in‐SNF MFD5. Of course, without a randomized controlled trial, we cannot establish causality or rule out potential physician biases which may underlie physician MFD5 choice. For the payor, proper transition to lower acute care site promotes more efficient targeted interventions appropriate for the needs of the patient. In this setting, meaningful interventions can be applied and evaluated for cost reduction and improved quality of care.

Our study is limited by lack of complete death or vintage information, as we were unable to secure access to CMS‐2746 death notification or CMS‐2728 dialysis initiation forms. CMS policy restricts access to identified patient‐level data for research exclusively to nonprofit entities. Cause of hospitalization is not captured in a retrievable manner in our EMR systems, which will be the focus of a future quality initiative. Finally, information on home discharge was gathered over the period May 1, 2020–June 20, 2020, coincident with the COVID‐19 pandemic in the US, so our analysis may not be an accurate reflection of home transfers in nonpandemic times. Likewise, the ability of nurses to carry out the intended MFD5 treatment was impacted to some degree during the pandemic due to illness‐induced staff shortages.

Detailed referral data were not available to us. Our best estimate was that roughly 90% of dialytic episodes occurred in individuals receiving dialysis in a post‐acute care setting; 85% of which are comprised of prevalent ESRD and 15% (683/4510) incident ESRD. Ten percent of dialytic episodes occurred in long‐term SNF residents with prevalent ESRD.

We provide the first detailed clinical and administrative summary of a hidden, growing, and needy population of dialysis patients. Future efforts will focus on detailed clinical analysis of interventions and outcomes with enhanced data collection driven by our quality improvement initiatives.

## CONFLICT OF INTEREST

Allen M. Kaufman, Alice M. Hellebrand, and Jordan G. Ledvina are current employees of Dialyze Direct and hold stock and/or stock options in Dialyze Direct. Steven M. Kaplan is a current employee of Dialyze Direct. Nathan W. Levin is a consultant for Dialyze Direct and chair of its Medical Advisory Board. Eran Y. Bellin is a consultant epidemiologist for Dialyze Direct, and William T. Markis is a consultant research analyst and medical writer for Dialyze Direct.

## Supporting information


**Figure S1** Table of organization for clinical evaluation and care with description of care model
**Figure S2**: Logistic gates to program entry
**Figure S3**: Clinical gates to program entry
**Figure S4**: Cumulative 90‐day mortality during COVID‐19 pandemic and non‐pandemic periods (2018, 2019, 2020)
**Figure S5**: Nursing Home Context and Service Contact Time
**Table S1**: First laboratory and assorted findings on first admission
**Table S2**: Laboratory and assorted findings of all dialytic episodes
**Table S3**: Count of dialytic episodes occurring within quintiles of % dialysis sessions with intradialytic hypotension, stratified by quintile averaged pre‐hemodialysis systolic blood pressure
**Table S4**: Relative mortality hazard risk for Cox model** stratified on quintile averaged pre‐dialysis systolic blood pressure***
