# Supplementary material for: Epidemiology of nursing home dialysis patients—A hidden population
Source: Hemodial Int. 2021 Jun 16;25(4):548–59. doi: 10.1111/hdi.12943 (PMC8596662; doi:10.1111/hdi.12943)
Supplement: Supplementary file 1 — Figure S1 Table of organization for clinical evaluation and care with description of care model Figure S2: Logistic gates to program entry Figure S3: Clinical gates to program entry Figure S4: Cumulative 90‐day mortality during COVID‐19 pandemic and non‐pandemic periods (2018, 2019, 2020) Figure S5: Nursing Home Context and Service Contact Time Table S1: First laboratory and assorted findings on first admission Table S2: Laboratory and assorted findings of all dialytic episodes Table S3: Count of dialytic episodes occurring within quintiles of % dialysis sessions with intradialytic hypotension, stratified by quintile averaged pre‐hemodialysis systolic blood pressure Table S4: Relative mortality hazard risk for Cox model** stratified on quintile averaged pre‐dialysis systolic blood pressure*** [file HDI-25-548-s001.docx]

**Epidemiology of Nursing Home Dialysis Patients – a Hidden Population**

Eran Y Bellin, Alice M Hellebrand, Steven M Kaplan, Jordan G Ledvina, William T Markis, Nathan W Levin, Allen M Kaufman

**Supplementary Material Table of Contents**

**Supplemental Figure S1**: Table of organization for clinical evaluation and care with description of care model

**Supplemental Figure S2**: Logistic gates to program entry

**Supplemental Figure S3**: Clinical gates to program entry

**Supplemental Figure S4**: Cumulative 90-day mortality during COVID-19 pandemic and non-pandemic periods (2018, 2019, 2020).

**Supplemental Figure S5**: Nursing Home Context and Service Contact Time

**Supplemental Table S1.** First laboratory and assorted findings on first admission

**Supplemental Table S2.** Laboratory and assorted findings of all dialytic episodes

**Supplemental Table S3.** Count of dialytic episodes occurring within quintiles of % dialysis sessions with intradialytic hypotension, stratified by quintile averaged pre-hemodialysis systolic blood pressure

**Supplemental Table S4.** Relative mortality hazard risk for Cox model** stratified on quintile average pre-dialysis systolic blood pressure***


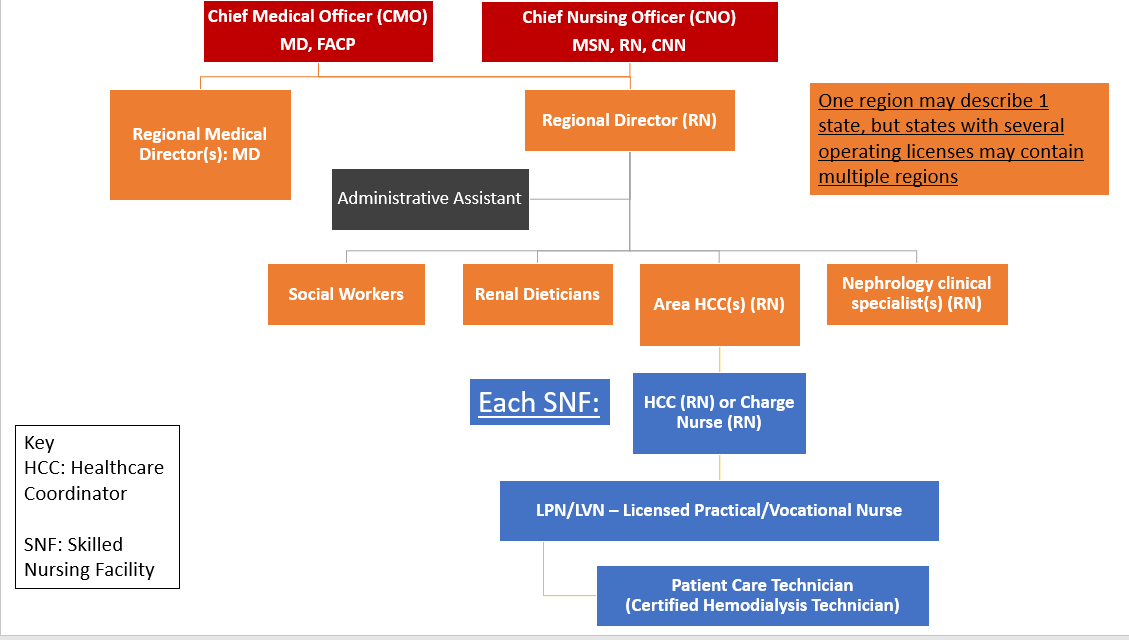


**Supplemental Figure S1: Table of organization for clinical evaluation and care.**

Prior to admitting patients and providing hemodialysis (HD) in the SNF, the centralized Dialyze Direct intake team coordinates a logistical and medical assessment gating process (Supplemental Figure 2, Supplemental Figure 3) encompassing expert nursing assessment, payor verification, staffing and resource allocation, oversight by an expert nephrologist, and medical record review followed by prescription orders by a credentialed local nephrologist of record. This local nephrologist assumes ongoing responsibility for dialysis management of the patient in the accepting SNF as implemented by Dialyze Direct’s on-site clinical team comprised of an RN home care coordinator and other professional caregivers and technicians. This team provides care under the guidance of personalized patient treatment protocols, centralized policy and procedures, telehealth technologies, and other material support from administrative Dialyze Direct nursing and nephrologist staff. They are aided by a regional care staff responsible for patient services across multiple regional SNFs.

**Supplemental Figure S2: Logistic gates to program entry**


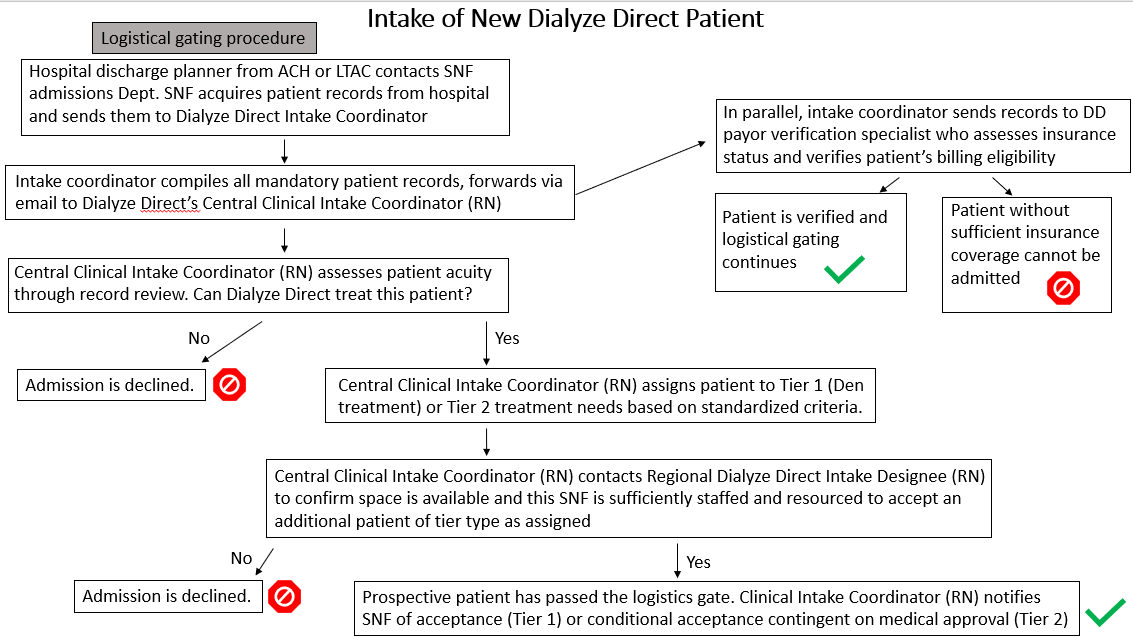


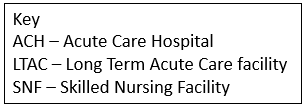


**Supplemental Figure S3: Clinical gates to program entry**


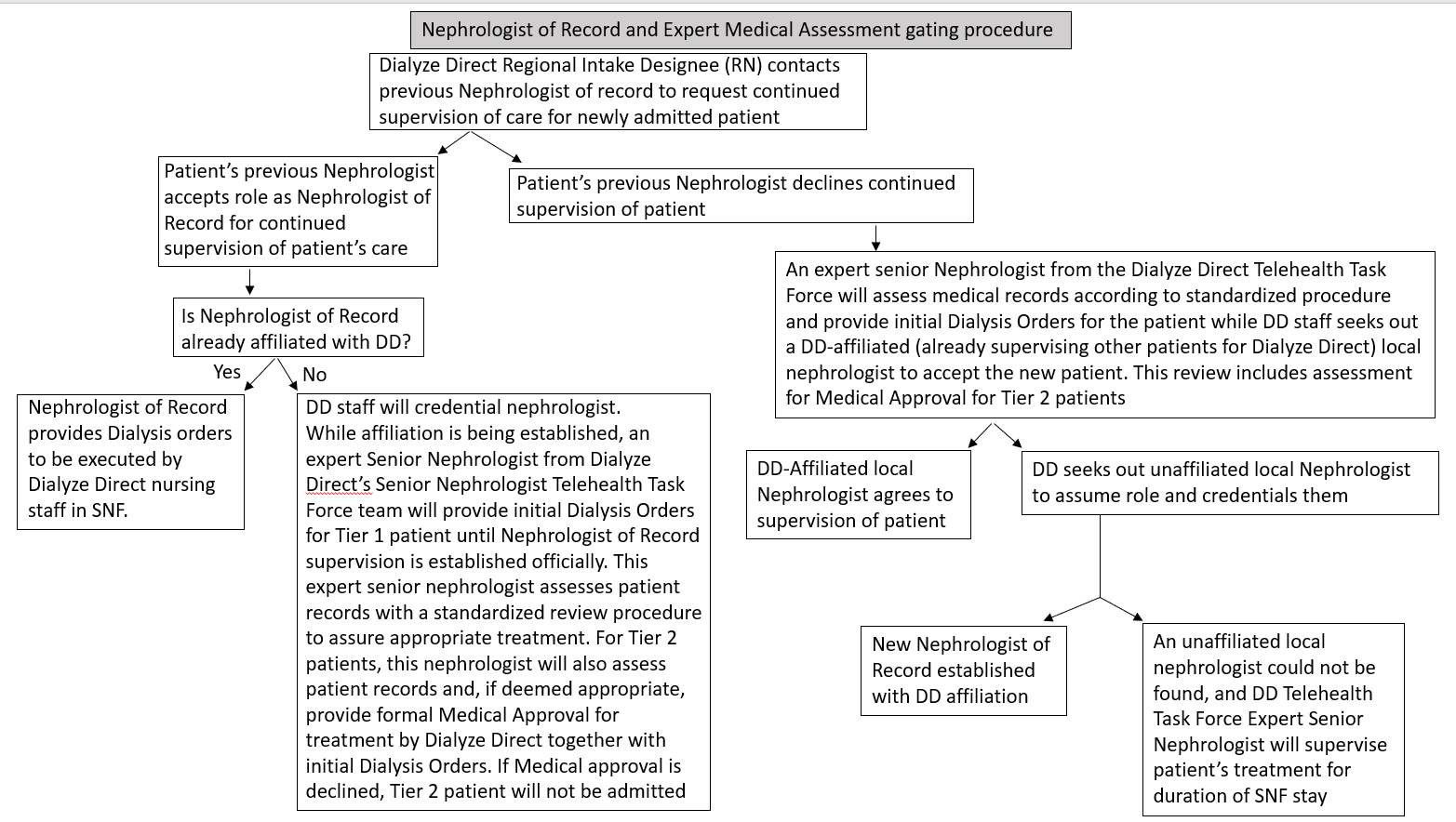


SNF, skilled nursing facility; DD, Dialyze Direct.


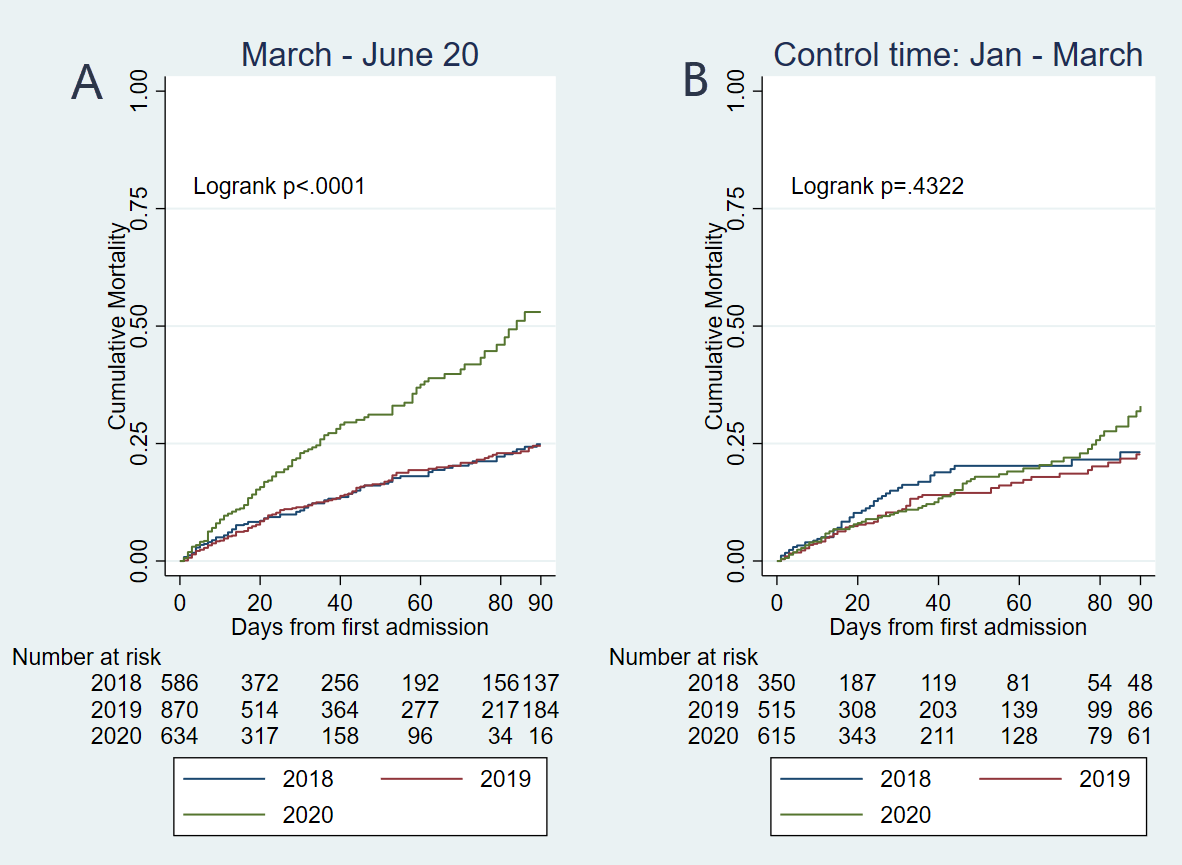


**Supplemental Figure S4: Cumulative 90-day mortality during COVID-19 pandemic and non-pandemic periods (2018, 2019, 2020).**

Comparison of 90-day mortality experience in patients admitted to a SNF during the COVID-19 pandemic and during two separate non-pandemic periods. (A) Cumulative 90-day mortality across three years limited to the calendar months within which COVID-19 was prevalent during 2020 in the study period (March – June 20th) and (B) Cumulative 90-day mortality across three years limited to the calendar months within which COVID-19 was not dramatically prevalent during 2020 (January-March). The first time a patient was admitted to a SNF in the relevant period and year was treated as time zero for a cumulative mortality analysis which was right-censored at 90 days in the absence of death. All available information from first SNF admission or subsequent SNF admission within the relevant period was used to calculate 90-day mortality incidence. A statistically significant increase in all-cause mortality was observed during the pandemic period of 2020 compared to like periods in previous years (A) (Kaplan-Meier log rank P<.0001). No difference in cumulative mortality was observed when comparing the pre-pandemic 2020 period to like periods in 2018 and 2019 (B) (Kaplan Meier log rank: p=.43).

This evidence suggests COVID-19 was responsible for a significant increase in all-cause mortality in this population of dialyzed nursing home residents during 2020. We unfortunately do not have access to death certificate data, so we cannot ascertain cause of death for more detailed analysis. In addition, in the early period of the COVID-19 pandemic, testing was not easily available nor uniformly applied. This is compounded by the uncertainty of ascertaining death due to COVID-19 vs. death with COVID-19. All our analyses of death in the body of the manuscript therefore refer to all-cause mortality as determined by review of the Electronic Medical Records maintained by Dialyze Direct staff.

These two mortality graphs were first presented in a poster, referenced: Kaufman AM, A Hellebrand, SM Kaplan, J Ledvina, NW Levin, EY Bellin: Canaries in the Coal Mine: Nursing Home Dialysis Patients as Sentinels during COVID [Abstract]. J Am Soc Nephrol 31, 2020: PO0716

**
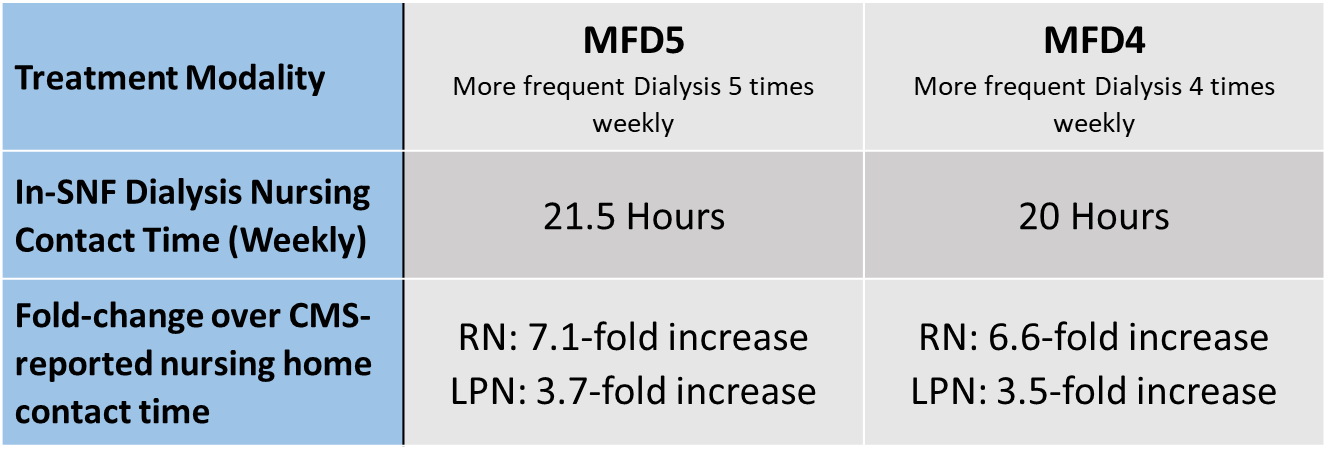
**

**Supplemental Figure S5: Nursing Home Context and Service Contact Time**

Dialyze Direct patients were served in 134 nursing homes in eight states. Total CMS-reported certified bed count for these nursing homes was 20,045. Average per resident per day contact time (hours) for the entire SNF population is reported for RN (median: 0.43; IQR: 0.38-0.47), LPN (median: 0.82; IQR: 0.77-0.86), and Nurse Aid (median: 2.1; IQR: 2.1-2.2). (All nursing home context data obtained from CMS Nursing Home Compare at <https://data.medicare.gov/widgets/4pq5-n9py>). In contrast, Dialyze Direct provided MFD5 patients with 21.5 hours per week contact time: 2.8 hours during dialysis, 45 minutes pre-HD assessment and put-on time, 45 minutes post-HD assessment, take-off, and hand-off time. For MFD4 patients there were 3.5 hours per HD with the same onset and take-off time, resulting in a net of 20 hours per week. The RN, LPN, and Patient Care Technician (PCT) provide direct patient care and spend 100% of their time with patient contact, with the RN providing direct oversight of the PCT and LPN during treatment hours. When provided MFD5, in-SNF dialysis patients therefore receive a 7-fold increase in RN and 3.7-fold increase in LPN median contact time per week compared to other SNF patients.

MFD5, more frequent dialysis five times weekly; MFD4, more frequent dialysis four times weekly; CMS, Center for Medicare and Medicaid Services; HD, hemodialysis.

**Supplemental Table S1.** First laboratory and assorted findings on first admission

|  | N | p5 | p10 | p25 | Median | p75 | p95 |
| --- | --- | --- | --- | --- | --- | --- | --- |
| Hematocrit (%) | 4430 | 23.1 | 24.5 | 26.7 | 29.5 | 32.7 | 37.9 |
| Albumin (g/dl) | 4431 | 2.2 | 2.4 | 2.7 | 3.1 | 3.4 | 3.9 |
| Calcium (mg/dl) | 4421 | 7.6 | 7.8 | 8.3 | 8.8 | 9.3 | 10.2 |
| Calcium Corrected (mg/dl) | 4375 | 8.5 | 8.7 | 9.1 | 9.5 | 9.9 | 10.8 |
| Phosphorus (mg/dl) | 4418 | 2.4 | 2.8 | 3.6 | 4.5 | 5.6 | 7.6 |
| Calcium Phos Product | 4418 | 20.2 | 24.0 | 31.0 | 39.2 | 49.4 | 69.0 |
| Ca Phos Product Corrected | 4371 | 22.7 | 26.7 | 34.0 | 42.6 | 53.0 | 73.3 |
| Creatinine (mg/dl) | 4438 | 2.4 | 2.9 | 4.0 | 5.5 | 7.3 | 10.5 |
| Sodium (mg/dl) | 4419 | 128 | 130 | 133 | 136 | 138 | 142 |
| Ferritin (ng/ml) | 4421 | 111 | 196 | 440 | 911 | 1396 | 2343 |
| Iron (µg/ml) | 4418 | 17 | 21 | 29 | 41 | 59 | 103 |
| IronSaturation (%) | 4386 | 9 | 11 | 16 | 22 | 31 | 55 |
| SystolicBP (mmHg) (first) | 4499 | 91 | 99 | 111 | 127 | 146 | 178 |
| DiastolicBP (mmHg) (first) | 4499 | 46 | 51 | 58 | 67 | 78 | 94 |
|  |  |  |  |  |  |  |  |

p, percentile (.01, .05, .10, .25, .5, .75, .95); N, number of observations; Ca, Calcium; Phos, Phosphate.

**Supplemental Table S2.** Laboratory and assorted findings of all dialytic episodes

|  | N | p5 | p10 | p25 | Median | p75 | p95 |
| --- | --- | --- | --- | --- | --- | --- | --- |
| Hematocrit (%) | 9177 | 22.7 | 24.2 | 26.5 | 29.3 | 32.5 | 37.9 |
| Albumin (g/dl) | 9183 | 2.2 | 2.4 | 2.7 | 3.1 | 3.5 | 4.0 |
| Calcium (mg/dl) | 9168 | 7.6 | 7.9 | 8.3 | 8.8 | 9.3 | 10.2 |
| Calcium Corrected (mg/dl) | 9111 | 8.5 | 8.7 | 9.1 | 9.5 | 9.9 | 10.8 |
| Phosphorus (mg/dl) | 9156 | 2.3 | 2.7 | 3.5 | 4.4 | 5.5 | 7.6 |
| Calcium Phos Product | 9164 | 19.4 | 23.2 | 30.2 | 39.0 | 49.0 | 68.0 |
| Ca Phos Product Corrected | 9107 | 22.0 | 26.0 | 33.0 | 42.0 | 52.2 | 72.7 |
| Creatinine (mg/dl) | 9195 | 2.47 | 2.97 | 3.97 | 5.55 | 7.44 | 10.68 |
| Sodium (mg/dl) | 9166 | 128 | 130 | 133 | 136 | 138 | 142 |
| Ferritin (ng/ml) | 9168 | 163 | 271 | 572 | 1002 | 1447 | 2363 |
| Iron (µg/ml) | 9162 | 18 | 22 | 30 | 43 | 61 | 105 |
| IronSaturation (%) | 9081 | 10 | 12 | 17 | 23 | 32 | 56 |
| SystolicBP (mmHg) (first) | 9233 | 92 | 99 | 111 | 128 | 148 | 179 |
| DiastolicBP (mmHg) (first) | 9233 | 46 | 51 | 59 | 68 | 78 | 95 |
|  |  |  |  |  |  |  |  |

p, percentile (.01, .05, .10, .25, .5, .75, .95); N, number of observations; Ca, Calcium; Phos, Phosphate.

**Supplemental Table S3.** Count of dialytic episodes occurring within quintiles of % dialysis sessions with intradialytic hypotension, stratified by quintile averaged pre-hemodialysis systolic blood pressure

| **Quintile % IDH* per dialytic episode** | **Quintile average pre-HD systolic Blood Pressure (per dialytic episode)** | | | | | **Total** |
| --- | --- | --- | --- | --- | --- | --- |
|  | 72-111.5 mmHg | 111.5-122.5 mmHg | 122.5-133.5 mmHg | 133.5-147.8 mmHg | ≥147.8 mmHg |  |
| **0 -10%** | 273 (15%) | 604 (33%) | 883 (48%) | 1,056 (58%) | 1,193 (65%) | 4.009 (44%) |
| **10-25%** | 134 (7%) | 379 (21%) | 392 (21%) | 338 (18%) | 303 (17%) | 1,546 (17%) |
| **25-40%** | 213 (12%) | 312 (17%) | 225 (12%) | 212 (12%) | 164 (9%) | 1,126 (12%) |
| **40-75%** | 577 (32%) | 379 (21%) | 252 (14%) | 176 (10%) | 116 (6%) | 1,500 (16%) |
| **75-100%** | 630 (34%) | 154 (8%) | 75 (4%) | 47 (3%) | 52 (3%) | 950 (10%) |
| **Total** | 1,827 | 1,828 | 1,827 | 1,829 | 1,828 | 9,139 (100%) |

*Percent of dialyses during a nursing home stay with at least one episode of intradialytic hypotension. Data are presented as *n* (%). HD, hemodialysis.

**Supplemental Table S4.** Relative mortality hazard risk for Cox model** stratified on quintile averaged pre-dialysis systolic blood pressure***

| **Quintile %IDH per dialytic episode*** | **Quintile average pre-HD systolic Blood Pressure (per dialytic episode)** | | | | |
| --- | --- | --- | --- | --- | --- |
|  | 72-111.5 mmHg | 111.5-122.5 mmHg | 122.5-133.5 mmHg | 133.5-147.8 mmHg | ≥147.8 mmHg |
| 0 -10% | 2.7 (1.3-5.6) | 2.0 (1.3-3.1) | 1.5 (1.0-2.2) | 1.3 (0.8-2.0) | 1.6 (1.0-2.6) |
| 10-25% | 1.0 | 1.0 | 1.0 | 1.0 | 1.0 |
| 25-40% | 2.0 (1.0-4.1) | 2.0 (1.2-3.3) | 1.6 (1.0-2.6) | 1.4 (0.8-2.5) | 1.6 (0.8-3.0) |
| 40-75% | 2.3 (1.2-4.4) | 2.4 (1.5-3.7) | 1.7 (1.1-2.7) | 0.9 (0.4-1.8) | 1.3 (0.6-3.1) |
| 75-100% | 3.8 (2.0-7.2) | 3.7 (2.1-6.6) | 6.1 (3.4-11.0) | 1.9 (0.4-8.0) | 22.0 (10.0-46.0) |

* Percent of dialyses during a nursing home stay with at least one episode of intradialytic hypotension.

** Cox model includes: Age, % IDH, sodium, albumin, creatinine, gender, race/ethnicity, and post-hemodialysis systolic blood pressure > pre-hemodialysis systolic blood pressure (% of dialyses).

*** Baseline mortality hazard applied to dialytic episodes with 10-25% dialysis sessions with intradialytic hypotension.

Data presented as hazard ratio (95% confidence interval).
